# Mycobacterial Cell Wall Stimulant in the Treatment of Squamous Cell Carcinoma: A Case Series Regarding Treatment in Equine, Bovine and Caprine Patients

**DOI:** 10.3389/fvets.2021.635800

**Published:** 2021-08-05

**Authors:** Jennifer Halleran, Katie Yau, Jillian Paegelow, Robert Streeter, Derek Foster

**Affiliations:** ^1^Department of Population Health and Pathobiology, College of Veterinary Medicine, North Carolina State University, Raleigh, NC, United States; ^2^Department of Veterinary Clinical Sciences, College of Veterinary Medicine, Oklahoma State University, Stillwater, OK, United States

**Keywords:** immunotherapy, squamous cell carcinoma, ruminant, neoplasia, equine

## Abstract

Squamous cell carcinoma (SCC) is a common dermatological neoplasia found in large animal species. Treatment options, such as surgery and cryotherapy may be difficult or not feasible. Alternative therapies, such as immunomodulating drugs, can potentially be used for companion large animals. The hypothesis of the following retrospective study is: following multiple intravenous and intralesional injections of a mycobacterial cell wall stimulant (MCW) regression of SCC in equine, bovine and caprine patients will be observed. In this observational-retrospective case series, patients included are 2 bovine, 2 caprine and 3 equine patients. The medical records at two different teaching veterinary hospitals were searched for cases with a positive histopathological diagnosis of squamous cell carcinoma that were subsequently treated with MCW, as either the sole therapy, or in conjunction with other therapies. Seven cases were included in this retrospective study. The median duration of therapy was 56.5 days, with 3 of the 7 patients being euthanized. Significant complications were seen in 3/7 patients. Repeated injections of a MCW may lead to reduction in lesion size of SCC in some cases, but long-term resolution is unlikely and the risk of significant complications is high; due to limited sample size and the variety in species, it is difficult to conclude if MCW is an effective therapy for SCC.

## Introduction

Squamous cell carcinoma (SCC) is a neoplasia of epithelial origin with certain predilection sites depending upon the species afflicted. Regardless of the species, however, several common factors may contribute to its development, such as genetic susceptibility, age and ultraviolet light exposure (1–5). Ocular squamous cell carcinoma (OSCC) is the most common malignant neoplasia of beef cattle, causing significant economic losses to cattle producers (1). Predilection sites include the lateral conjunctiva, corneolimbal junction, lower eyelid, nictitating membrane and medial canthus (1, 2). Lesions in the lower eyelid and nictitating membrane tend to be more aggressive and the neoplasm has the ability to metastasize to regional lymph nodes and the lungs (1). Surgical excision with clean margins can be curative. Non-resectable lesions are very difficult to manage and treatment modalities for such cases are limited to tertiary referral hospitals. Similar to OSCC in cattle, squamous cell carcinoma (SCC) is the most common malignant skin tumor in horses, comprising 7–37% of them (6). As a frequently diagnosed tumor, SCC has a significant impact on the health and welfare of affected horses (6). The most common treatment option is wide surgical excision of the tumor, which is not always practical or effective (6). In goats, SCC is characterized by its invasive nature and risk factors shared by other species. It is most commonly found on the perineum (7, 8). Studies have suggested other risk factors in goats including a predisposition for females and smaller breed animals (9). Combining surgical treatment with an immunotherapy has the potential to improve the recurrence rate associated with these tumors.

Immunotherapy may be a viable treatment option when surgical excision cannot be performed. Immunotherapy enhances the immune system to cause tumor regression. There are many different types of immunotherapy present in human and veterinary medicine, some of which have been used previously to treat OSCC-saline-phenol extract prepared from OSCC, bacillus Calmette-Guerin for intralesional injections and IL-2 therapy (10–12). There is a Mycobacterium cell wall (MCW)^1^ extract labeled for canine mixed mammary gland adenocarcinoma and equine sarcoids (13). The MCW has immunomodulatory effects, where it promotes the production of anti-cancer cytokines and lymphocytes (13). It has direct and indirect effects on the neoplastic cells. Directly, it can induce apoptosis on the neoplastic cells (13). Indirectly, the MCW stimulates dendritic cells, macrophages, and monocytes to produce anti-cancer cytokines-IL-6, IL-8, IL-10, IL-12, IL-18 and TNFα (13). Through this, natural killer cells are stimulated and the combination allows for induction of apoptosis and cell lysis.

This report describes the use of the MCW in two mature cattle with non-resectable OSCC, three horses with SCC of the peri-anal region, preptial sheath, and vulva and two goats with SCC on the perineum. This is a limited case series with the hopes of utilizing the MCW in future SCC cases to further our understanding of treatment with this agent.

## Materials and Methods

Cases utilizing MCW therapy were searched at both Oklahoma State University Boren Veterinary Medical Teaching Hospital and North Carolina State University Veterinary Teaching Hospital. Case searches were conducted using the online computer system Universal Veterinary Information System (UVIS, Atlanta GA). Cases were found by searching the diagnosis of squamous cell carcinoma and were subsequently reviewed. Inclusion criteria for this retrospective case series included a histopathological diagnosis of squamous cell carcinoma followed by MCW therapy. The MCW therapy may have been the sole therapy or administered in conjunction with other therapies, such as surgical resection or cryotherapy.

After identifying appropriate cases, the medical records were reviewed. Signalment, presenting physical exam findings, histopathologic diagnosis, location of lesion, treatment and outcome were all recorded and analyzed.

## Results

**Figure 1:**
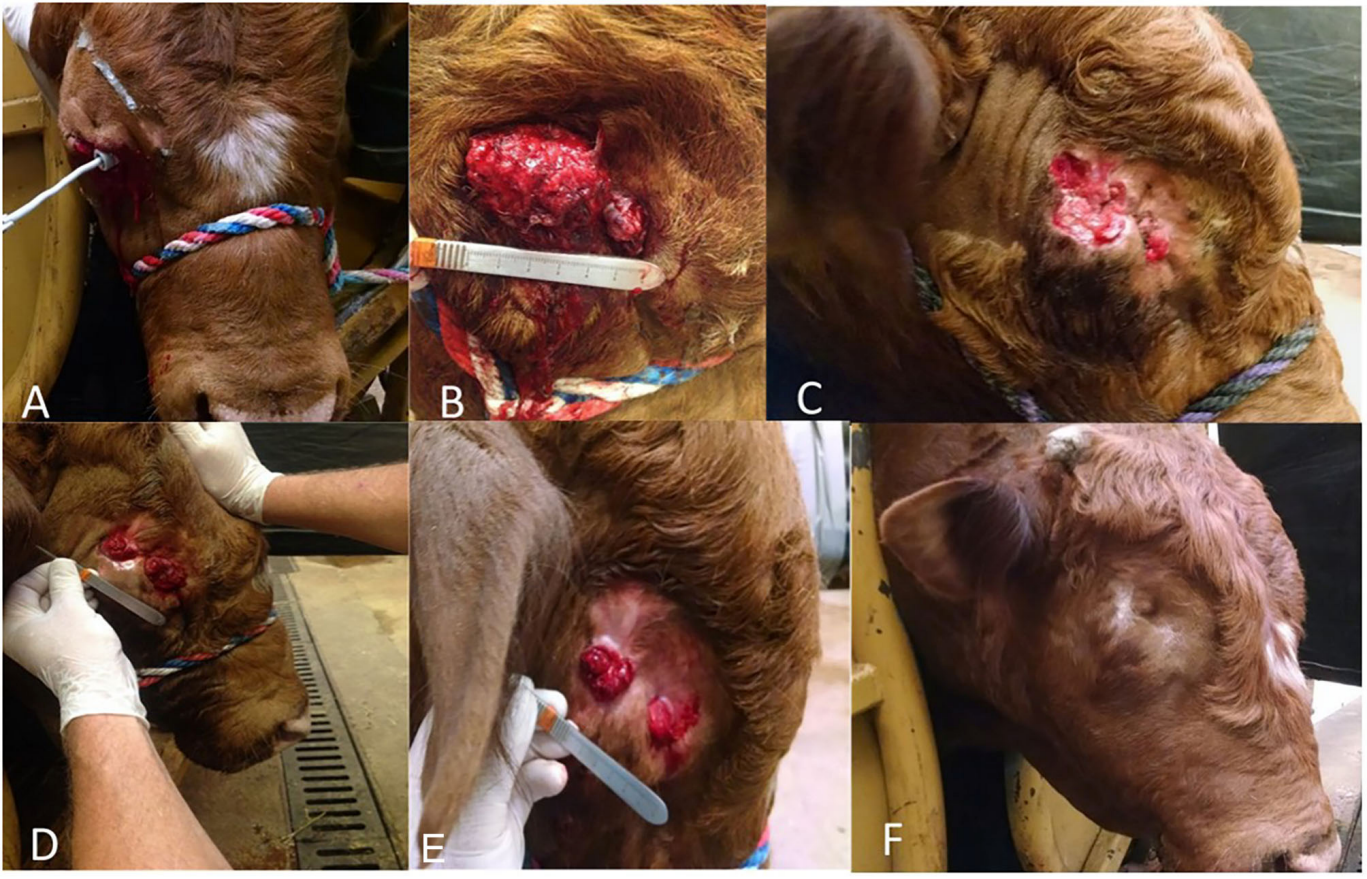
Three-year-old bucking bull diagnosed with OSCC. **(A–F)** reflect case progression. **(A)** Two weeks post-extirpation. **(B)** Two months post-extirpation. **(C)** Three months post-extirpation. **(D)** Four months post-extirpation. **(E)** Five months post-extirpation. **(F)** Eight months post-extirpation.

**Figure 2:**
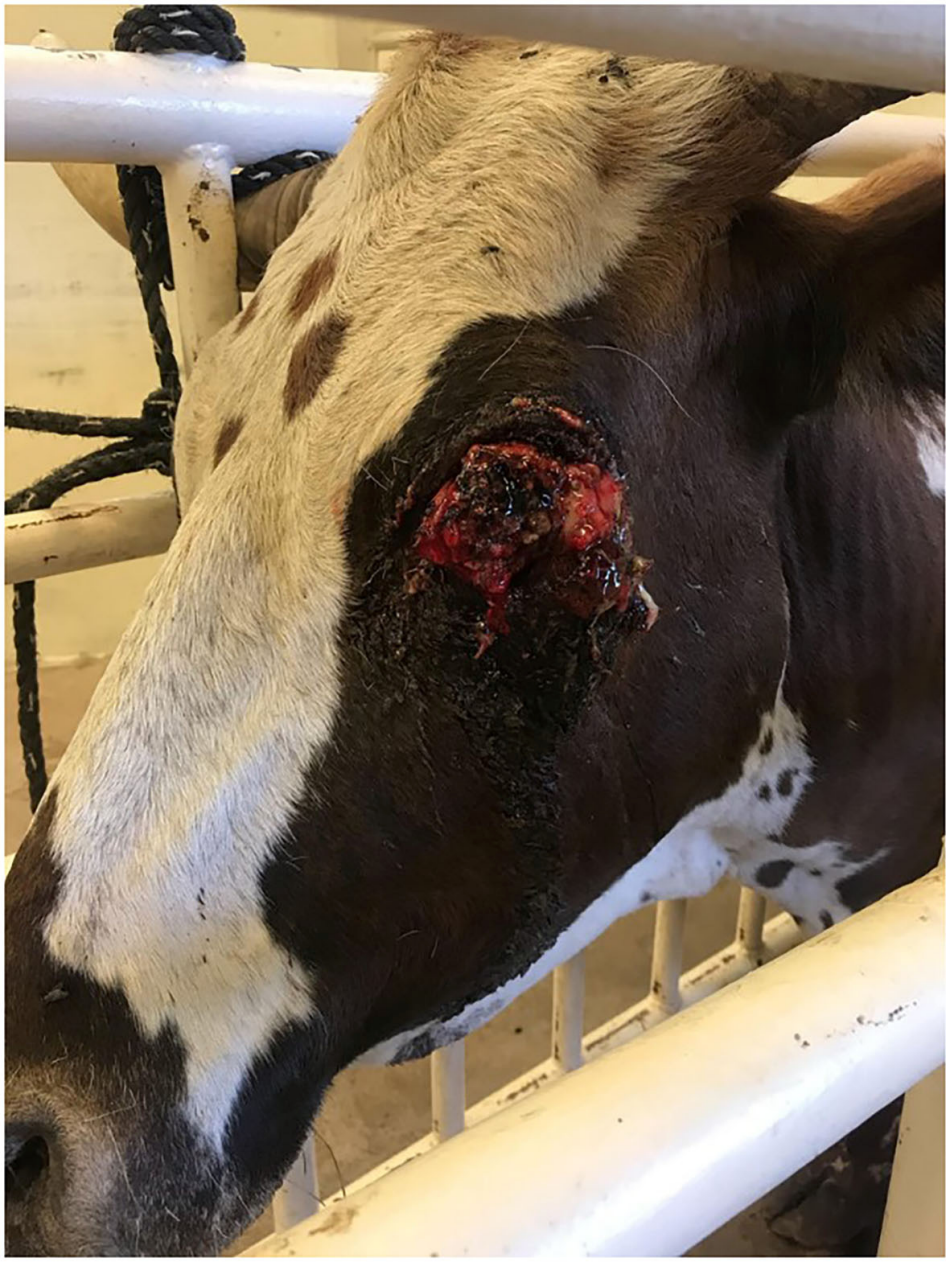
Twelve-year-old Longhorn cow diagnosed with OSCC.

**Figure 3:**
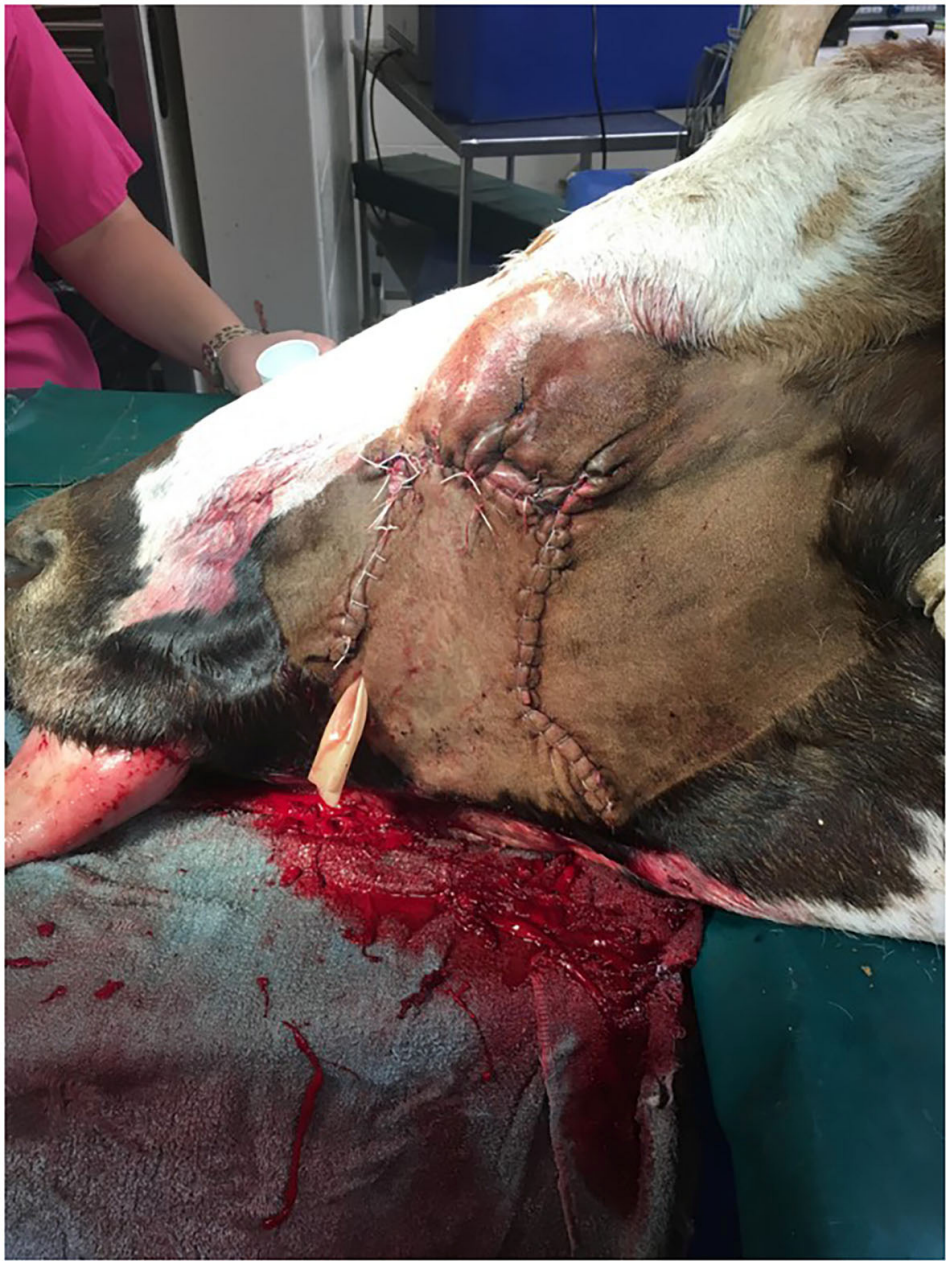
Longhorn cow post-extirpation with sliding H-plasty. Drain placement is in ventral aspect of the incision.

**Figure 4:**
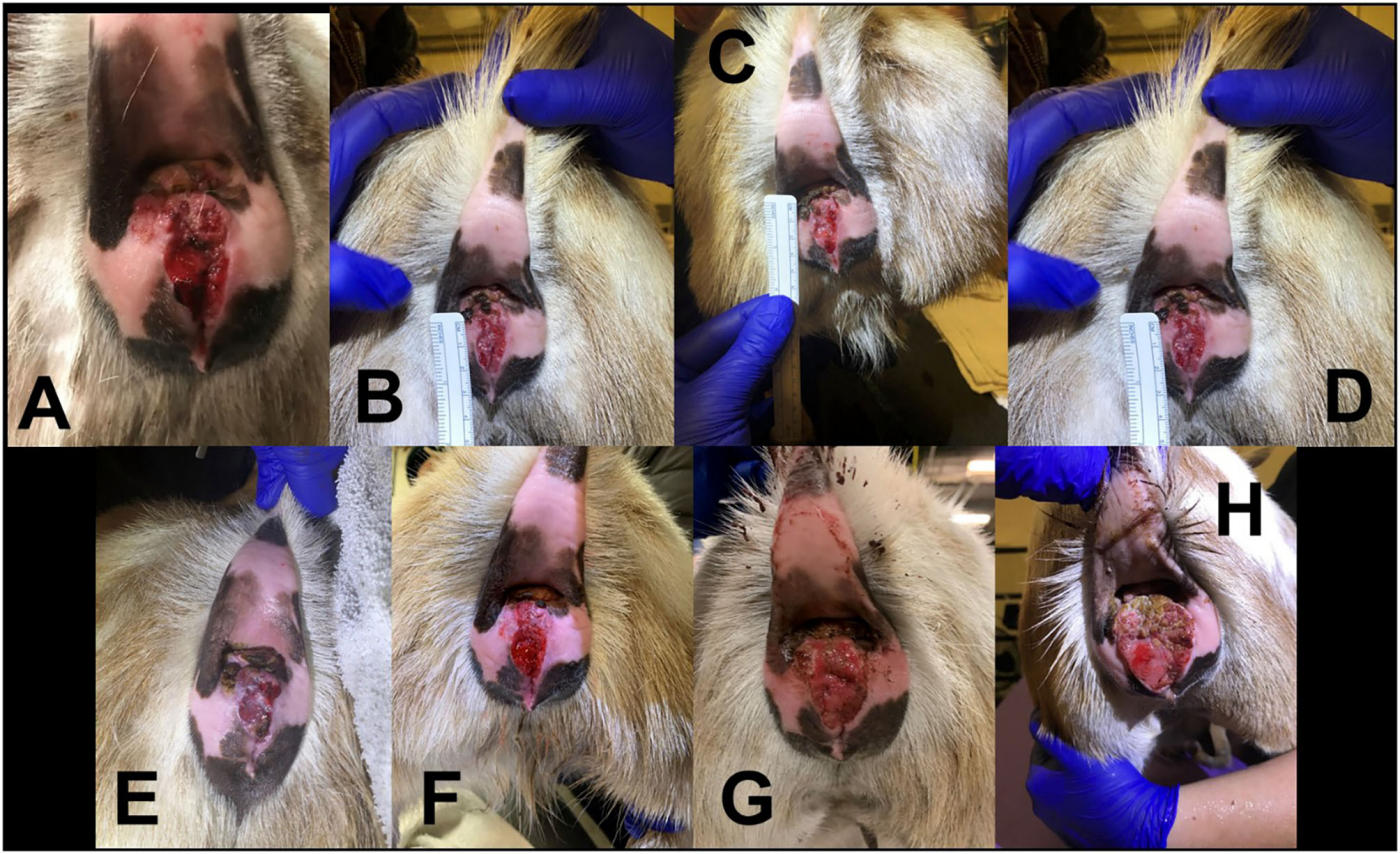
Progression of 3.5 year old Nigerian Dwarf doe perineal SCC. **(A)** Initial presentation of lesion prior to MCW therapy. **(B)** Second treatment of MCW, 1 week from presentation. **(C)** Third treatment, 2 weeks from presentation. **(D)** Fourth treatment, 3 weeks from presentation. **(E)** Fifth treatment, 4 weeks from presentation. **(F)** Six weeks from presentation. **(G)** Nineteen weeks from presentation. **(H)** Thirty-three weeks from presentation.

**Figure 5:**
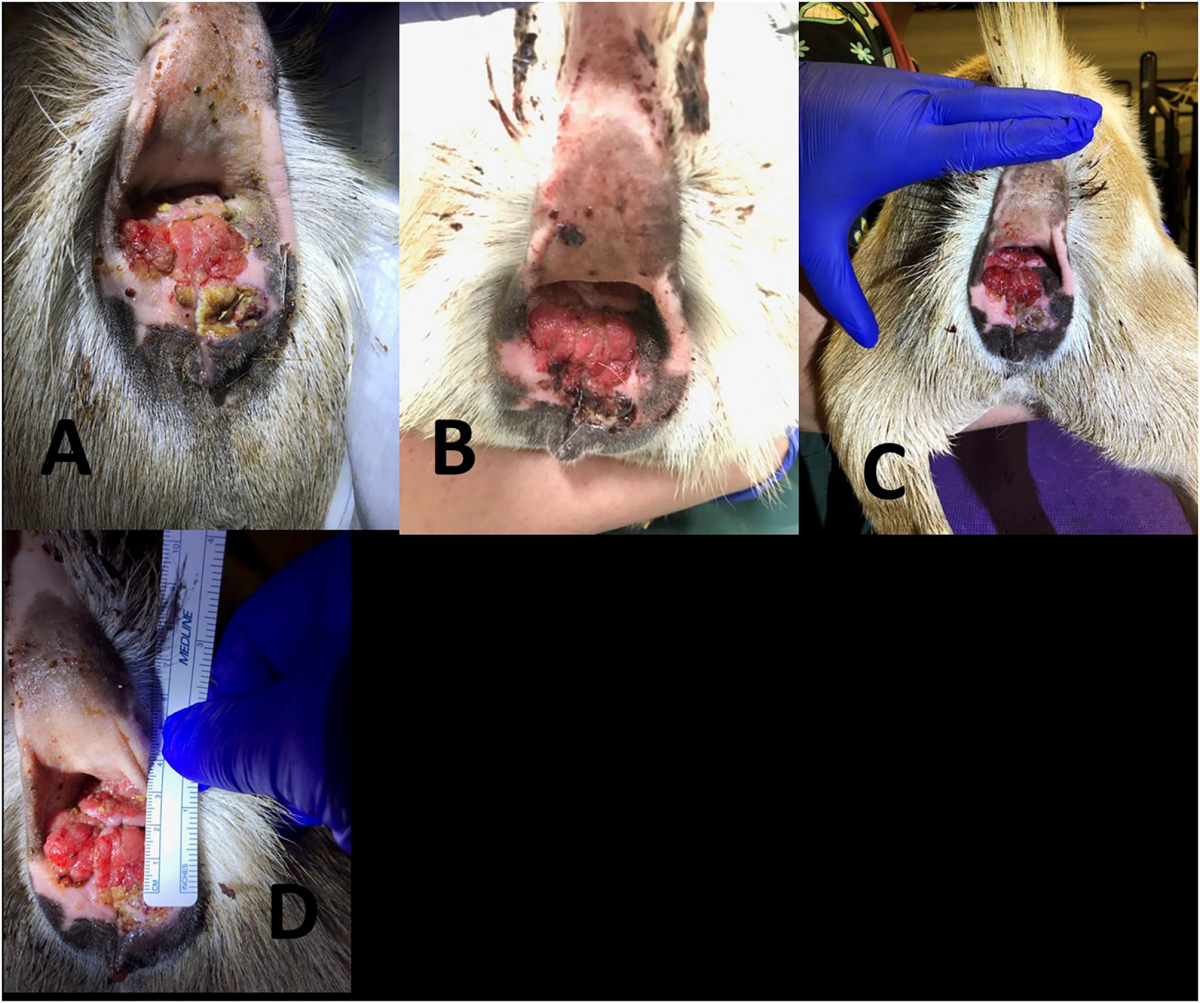
Progression of 7 year old Nigerian Dwarf doe perineal SCC. **(A)** Prior to the first treatment with MCW. **(B)** Second treatment, 1 week after the first therapy. **(C)** Third treatment, 2 weeks after the first therapy. **(D)** Fourth treatment, 3 weeks after the first therapy.

After review of the medical records, 7 cases were identified and included for further analysis. These cases included 2 bovine (Figures 1–3), 3 equine and 2 caprine patients (Figures 4, 5). Signalment and treatment summary can be found in Table 1. Each lesion was diagnosed positive as squamous cell carcinoma based on a biopsy of the affected tissue. Location of the lesion varied per species. Both cattle were afflicted with OSCC, both goats had perineal SCC and the equine patients had lesions present as a peri-anal mass, lesion on the penis, prepuce and sheath and the vulvar region.

**Table 1:** This table demonstrates the 7 cases that are included for review of the use of Immunocidin in the treatment of squamous cell carcinoma.

**Signalment**	**Lesion location**	**Initial size of lesion**	**Treatment**	**Dose of immunocidin**	**Final size of lesion recorded**	**Duration of treatment**	**Outcome**
3 year old Bucking Bull	Medial canthus of the right eye	N/A	Right Eye transpalpebral extirpation; Cyrotherapy; Immunocidin therapy (4 intralesional doses)	1–6 mL	N/A	120 days	No reoccurrence for 2 years, euthanized when reoccurrence was noted
12 year old Longhorn Cow	Entire left eye	N/A	Left eye transpalpebral extirpation and sliding H-plasty advancement flap; Immunocidin (3 intralesional doses)	2.5–5 mL	N/A	28 days	Passed away at home 90 days post extirpation
27 year old Paint Gelding	Peri-anal mass	12.7 cm in diameter	Surgical removal; Immunocidin therapy (2 intralesional doses)	10 mL	7 cm x 7 cm	35 days	Discharged
17 year old Paint Gelding	Penis, prepuce and sheath	N/A	Surgical removal; Immunocidin therapy (5 intralesional doses); Cisplatin bead therapy	2.5 mL	N/A	—	Euthanized (colic)
9 year old Paint Mare	Vulva	3 masses-12 cm × 7 cm; 2 cm × 2 cm; 2 cm × 5 cm	Surgical removal; Immunocidin therapy (4 intralesional doses)	15–20 mL	3 masses-3 cm; 1 cm; 2 cm	78 days	Discharged
3.5 year old Nigerian Dwarf Goat doe	Perineum	5 cm × 1.2 cm	Immunocidin therapy (5 intravenous doses, 1 intralesional dose)	Immunocidin 0.25 mL diluted in 3 mL given intralesional and 0.25 mL diluted in 10 mL given IV		270 days	Re-biopsy and retreat
5 year old Nigerian Dwarf Goat doe	Perineum	4 cm × 4 cm	Immunocidin therapy (4 intravenous doses, 1 intralesional dose)	Immunocidin 0.25 mL diluted in 3 mL given intralesional and 0.25 mL diluted in 10 mL given IV	3 cm × 4 cm	30 days	Discharged

In addition to MCW, surgical therapy and mass debulking were the most common ancillary treatment options. One bull did have cryotherapy performed prior to commencing MCW therapy. The dose of MCW varied from case to case and was based upon the clinician in charge of the case. The bovine patients received only intralesional therapy that was not diluted and administered in a grid-like pattern. The equine patients' dose ranged from 2.5 mL (prepuce lesion) up to 20 mL. This was administered circumferentially around the periphery of the lesion. The caprine patients received both intravenous (0.25 mL diluted in 10 mL) and intralesional (0.25 mL diluted in 3 mL) doses. Only 4 of the 7 cases had initial measurements recorded of the lesion size. Of the 4 cases with lesion sizes recorded, 3 of the lesions decreased in size over time; one is currently still receiving therapy. The median duration of therapy was 56.5 days (range: 28–270 days).

Three patients present in this case study either died or were euthanized. The bucking bull was euthanized due to recurrence of the tumor after a 2-year remission period. The Longhorn cow died 90 days post extirpation at home with no necropsy performed. Although no external lesions were observed at time of death, the cow was reported by the client to have had a head tilt and difficulty chewing. The gelding with preputial lesions was euthanized due to colic; prior to euthanasia, it was noted the prepuce lesions had multiple draining tracts present.

Although the other cases were discharged and are still alive at this time, complications were noted in two of the four, and resolution of disease was not seen. The Paint mare developed ulcerative lesions with draining tracts along the vulva about a month into therapy. Although the site did heal after surgical excision of the draining tracts, the mare developed limb and inguinal edema; at that time, it was decided to discontinue MCW therapy and treat with surgical debulking. The 3.5 year old Nigerian dwarf goat at first responded well to MCW therapy with a shrinking mass; however, at day 256 into therapy, the mass was larger than at presentation and a second biopsy confirmed SCC was still present. With confirmation of SCC on biopsy, MCW therapy was continued for the Nigerian dwarf doe. However, with repeated MCW therapy, the doe developed severe anaphylactic reaction after intralesional injection. The doe went into cardiac arrest and was resuscitated, but had significant muscle fasciculations present in the area of the intralesional therapy. The doe did recover, and a second attempt of intralesional MCW therapy was made; a less severe anaphylactic reaction was noted and the doe recovered without complication.

The older Paint gelding was discharged. The other Nigerian Dwarf doe is currently being monitored for changes in size and character of the lesion.

## Discussion

This retrospective study highlighted the use of MCW therapy in two bovine, three equine and two caprine patients whom had a positive histopathological diagnosis of squamous cell carcinoma. Treatment of SCC in large animals typically consists of surgery, cryotherapy or chemotherapy in horses. Despite these therapies, incomplete resection and regrowth commonly occurs, with the possibility of metastasis. The mycobacterial cell wall stimulant, used to treat other types of neoplasia was utilized here in seven cases to assess its effectiveness at treating SCC in a wide array of patients to observe if it could be a potential treatment option.

In summary, the median duration of treatment was 56.5 days. Most patients received at least 3 doses of MCW, both intravenously and intralesionally. Four cases had a record of initial lesion measurement; at the end of therapy, 3 of these 4 cases demonstrated a decrease in lesion size. However, significant complications were seen in several cases. Two of the equine patients developed draining tracts and inflammation secondary to intralesional MCW injections. One goat, although initially demonstrated a decrease in lesion size, now has a large lesion that is still histopathologically a SCC lesion and experienced anaphylaxis due to repeated MCW injections. According the author's knowledge, this has not yet been reported in the scientific literature. Ultimately, three patients are deceased.

An unusual finding in both of the bovine cases was the ocular pigmentation present. It has been shown that circumocular apigmentation increases the risk of development of OSCC. The bull in this case had a completely pigmented head and the Longhorn cow had pigment around both of its eyes. There has been one other documented case of OSCC in a pigmented crossbred cow.^10^ In this case report, the cow was housed in a roofed area, with limited exposure to sunlight. The underlying etiology for this cow was not determined. The bucking bull and Longhorn were both kept outside on pasture; no other animals on the property had been afflicted with OSCC. The underlying etiology of this disease is multifactorial; perhaps these animals had a genetic susceptibility to the development of OSCC. Both caprine cases had a non-pigmented perineum.

The MCW is a chemotherapeutic drug, but the carcinogenic/mutagenic effects are not well-known. It is a USDA licensed product, meaning extra-label drug use of would be prohibited. The owners of food animals whom received MCW in this case series agreed to never allow them to enter the food chain, and therefore this SCC therapy would be recommended for patients of economic or sentimental value that too would not enter the food chain.

This is a small case series consisting of seven cases where MCW was used to treat SCC in large animal patients. Based on the cases presented here, although 4 cases did show a reduction in lesion size, and one had long term remission, complications were noted in 3/7 cases. More cases are needed to conclusively determine if this is an appropriate treatment option for SCC in companion large animals, though clinicians should be aware of the potential for significant complications. With such a small case series and diverse species group, accurate conclusions regarding the efficacy of MCW for treating SCC cannot currently be justified.

## Data Availability Statement

The original contributions presented in the study are included in the article/supplementary material, further inquiries can be directed to the corresponding author/s.

## Ethics Statement

Ethical review and approval was not required for the animal study because Restrospective Case Study. Written informed consent was obtained from the owners for the participation of their animals in this study.

## Author Contributions

JH, RS, and DF: study design and idea. JH, RS, KY, JP, and DF: writing and edit of manuscript. JH, KY, and JP: collection of cases. All authors contributed to the article and approved the submitted version.

## Conflict of Interest

NovaVive supplied some product (Immunocidin) for free for the treatment of the bovine and caprine patients. The authors declare that the research was conducted in the absence of any commercial or financial relationships that could be construed as a potential conflict of interest.

## Publisher's Note

All claims expressed in this article are solely those of the authors and do not necessarily represent those of their affiliated organizations, or those of the publisher, the editors and the reviewers. Any product that may be evaluated in this article, or claim that may be made by its manufacturer, is not guaranteed or endorsed by the publisher.

## Footnotes

^1^Immunocidin, NovaVive Inc., Napanee Canada 2020.
